# Dr. Alexander John Alliott

**Published:** 1910-09-10

**Authors:** 


The death is announced, at Sevenoaks, of Dr. Alexander John Alliott, in his sixty-third year. A scholar of Pembroke College, Cambridge, he took his B.A. in 1870, his M.R.C.S. in 1872, his M.B. in 1873, and graduated M.D. in 1880. He studied also at St. Thomas's and at Manchester, and his appointments included that of resident clinical assistant at the Bethlem Royal Hospital and later that of surgeon to the Hospital for Children with Hip Disease, and to the Cottage Hospital, Sevenoaks. He had been assistant-medical officer to the Bedford County Lunatic Asylums, and was a member of the Medico-Psychological Society.

